# Supplementary material for: Genome-Wide Identification and Analysis of the MADS-Box Gene Family in Almond Reveal Its Expression Features in Different Flowering Periods
Source: Genes (Basel). 2022 Sep 29;13(10):1764. doi: 10.3390/genes13101764 (PMC9601849; doi:10.3390/genes13101764)
Supplement: Supplementary file 1 [file genes-13-01764-s001.zip › Supplementary Figures.pdf]

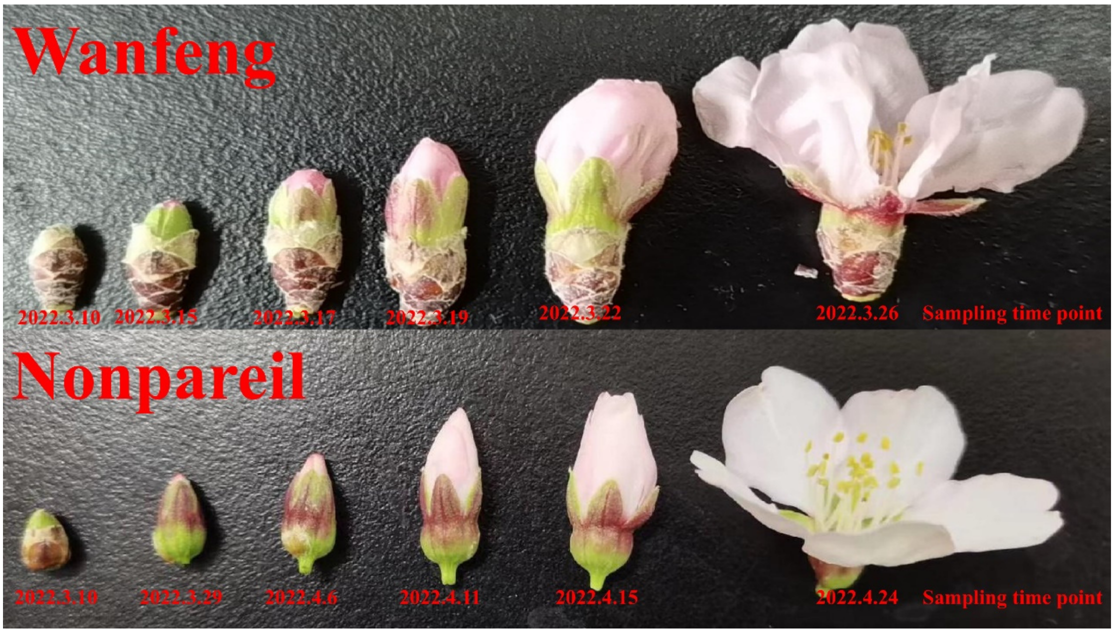

Figure S1 Six flowering periods of 'Wanfeng' and 'Nonpareil' almonds

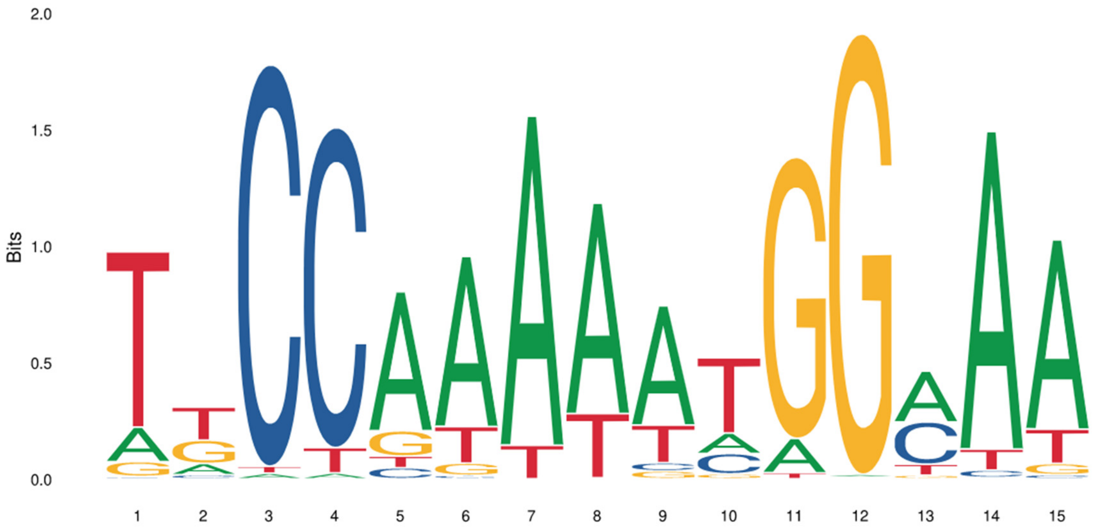

Figure. S2 The consensus motif of the MIKC DNA binding site from the JASPA CORE database
